# Supplementary material for: Empirical methods for controlling false positives and estimating confidence in ChIP-Seq peaks
Source: BMC Bioinformatics. 2008 Dec 5;9:523. doi: 10.1186/1471-2105-9-523 (PMC2628906; doi:10.1186/1471-2105-9-523)
Supplement: Additional File 1 — A variety of html documents from the USeq web site detailing the available applications, their best usage, output file type descriptions, command line menus, etc. [file 1471-2105-9-523-S1.zip › USeqUserGuides/JavaDoc.zip!/JavaDoc/bioroot/antibody/AntibodyModify.html]

AntibodyModify


|  |  |  |
| --- | --- | --- |
| **Overview** \| **Package** \| **Class** \| **Use** \| **Tree** \| **Deprecated** \| **Index** \| **Help** | |  |
| **PREV CLASS**   **NEXT CLASS** | **FRAMES**    **NO FRAMES**     **All Classes** |  |
| SUMMARY: NESTED \| FIELD \| CONSTR \| METHOD | DETAIL: FIELD \| CONSTR \| METHOD |  |


---


## bioroot.antibody Class AntibodyModify

```
java.lang.Object
  javax.servlet.GenericServlet
      javax.servlet.http.HttpServlet
          bioroot.antibody.AntibodyModify
```

**All Implemented Interfaces:**: java.io.Serializable, javax.servlet.Servlet, javax.servlet.ServletConfig

---

``` public class AntibodyModify extends javax.servlet.http.HttpServlet ```

Servlet to modify antibodys using antibodyBeans referenced from a Session object.

**Author:**
:   nix

**See Also:**: Serialized Form

---

**Constructor Summary**

`AntibodyModify()`


| **Method Summary** | |
| --- | --- |
| `static java.lang.String` | `deleteAntibody(AntibodyBean ab, UserBean userBean, DBUtil bioRoot)`             Attempts to delete an antibody, if sucessful, returns a "" String, otherwise an error message. |
| `void` | `doGet(javax.servlet.http.HttpServletRequest request, javax.servlet.http.HttpServletResponse response)` |
| `void` | `doPost(javax.servlet.http.HttpServletRequest request, javax.servlet.http.HttpServletResponse response)` |
| `static boolean` | `subtractAndSetAntibodyBeans(AntibodyBean[] beans, javax.servlet.http.HttpSession session)`             Subtracts a bean from the AntibodyBean[] and resets it in antibodyBeansToEdit |
| `static java.lang.String` | `updateAntibody(AntibodyBean bean, UserBean userBean, DBUtil bioRoot)`             Attempts to update a AntibodyBean in the database, only checks that the name is unique if it has changed. |
| `static java.lang.String` | `updateAntibodyPreps(AntibodyBean bean, DBUtil bioRoot)`             Attempts to update the AntibodyPreps and UseDilutions in the database. |
| `static boolean` | `updateCommonAccessFields(AntibodyPrep[] preps, DBUtil bioRoot)`             Just update the commonly accessible fields in each antibodyPrep as well as the useDilutions. |

**Methods inherited from class javax.servlet.http.HttpServlet**

`service`

**Methods inherited from class javax.servlet.GenericServlet**

`destroy, getInitParameter, getInitParameterNames, getServletConfig, getServletContext, getServletInfo, getServletName, init, init, log, log`

**Methods inherited from class java.lang.Object**

`equals, getClass, hashCode, notify, notifyAll, toString, wait, wait, wait`

**Constructor Detail**

### AntibodyModify

```
public AntibodyModify()
```


**Method Detail**

### doGet

```
public void doGet(javax.servlet.http.HttpServletRequest request,
                  javax.servlet.http.HttpServletResponse response)
           throws javax.servlet.ServletException,
                  java.io.IOException
```

:   **Overrides:**: `doGet` in class `javax.servlet.http.HttpServlet`
:   **Throws:**: `javax.servlet.ServletException`: `java.io.IOException`

---


### doPost

```
public void doPost(javax.servlet.http.HttpServletRequest request,
                   javax.servlet.http.HttpServletResponse response)
            throws javax.servlet.ServletException,
                   java.io.IOException
```

:   **Overrides:**: `doPost` in class `javax.servlet.http.HttpServlet`
:   **Throws:**: `javax.servlet.ServletException`: `java.io.IOException`

---


### deleteAntibody

```
public static java.lang.String deleteAntibody(AntibodyBean ab,
                                              UserBean userBean,
                                              DBUtil bioRoot)
```

:   Attempts to delete an antibody, if sucessful, returns a "" String, otherwise an error message.
    Also deletes the associated antibody preps, use dilutions, strain and plasmid references.
    All associated files are deleted too.
    If you change the wording of the error messages, update them in the AntibodySpreadSheet yesDelete section.

---


### updateCommonAccessFields

```
public static boolean updateCommonAccessFields(AntibodyPrep[] preps,
                                               DBUtil bioRoot)
```

:   Just update the commonly accessible fields in each antibodyPrep as well as the useDilutions.

---


### updateAntibodyPreps

```
public static java.lang.String updateAntibodyPreps(AntibodyBean bean,
                                                   DBUtil bioRoot)
```

:   Attempts to update the AntibodyPreps and UseDilutions in the database.
    If the update is sucessful, the message will be "", otherwise an error message will be returned.

---


### updateAntibody

```
public static java.lang.String updateAntibody(AntibodyBean bean,
                                              UserBean userBean,
                                              DBUtil bioRoot)
```

:   Attempts to update a AntibodyBean in the database, only checks that the name is unique if it has changed.
    If the update is sucessful, the message will be "", otherwise an error message will be returned.

---


### subtractAndSetAntibodyBeans

```
public static boolean subtractAndSetAntibodyBeans(AntibodyBean[] beans,
                                                  javax.servlet.http.HttpSession session)
```

:   Subtracts a bean from the AntibodyBean[] and resets it in antibodyBeansToEdit


---


|  |  |  |
| --- | --- | --- |
| **Overview** \| **Package** \| **Class** \| **Use** \| **Tree** \| **Deprecated** \| **Index** \| **Help** | |  |
| **PREV CLASS**   **NEXT CLASS** | **FRAMES**    **NO FRAMES**     **All Classes** |  |
| SUMMARY: NESTED \| FIELD \| CONSTR \| METHOD | DETAIL: FIELD \| CONSTR \| METHOD |  |


---
